# Predicting Online Behavioural Responses to Transcranial Direct Current Stimulation in Stroke Patients with Anomia

**DOI:** 10.3390/life14030331

**Published:** 2024-03-01

**Authors:** Thomas M. H. Hope, Sasha Ondobaka, Haya Akkad, Davide Nardo, Katerina Pappa, Cathy J. Price, Alexander P. Leff, Jennifer T. Crinion

**Affiliations:** 1 Institute of Cognitive Neuroscience, University College London, London WC1N 3AZ, UKhaya.akkad.14@ucl.ac.uk (H.A.); a.leff@ucl.ac.uk (A.P.L.); j.crinion@ucl.ac.uk (J.T.C.); 2 Wellcome Centre for Human Neuroimaging, University College London, London WC1N 3AR, UK; c.j.price@ucl.ac.uk; 3 Department of Education, University of Roma Tre, 00185 Rome, Italy; davidenardo@gmail.com; 4 Department of Psychological Sciences & Health, University of Strathclyde, Glasgow G1 1XP, UK; katerina.pappa@strath.ac.uk

**Keywords:** neurostimulation, stroke, aphasia, prediction

## Abstract

Anomia, or difficulty naming common objects, is the most common, acquired impairment of language. Effective therapeutic interventions for anomia typically involve massed practice at high doses. This requires significant investment from patients and therapists. Aphasia researchers have increasingly looked to neurostimulation to accelerate these treatment effects, but the evidence behind this intervention is sparse and inconsistent. Here, we hypothesised that group-level neurostimulation effects might belie a more systematic structure at the individual level. We sought to test the hypothesis by attempting to predict the immediate (online), individual-level behavioural effects of anodal and sham neurostimulation in 36 chronic patients with anomia, performing naming and size judgement tasks. Using clinical, (pre-stimulation) behavioural and MRI data, as well as Partial Least Squares regression, we attempted to predict neurostimulation effects on accuracies and reaction times of both tasks. Model performance was assessed via cross-validation. Predictive performances were compared to that of a null model, which predicted the mean neurostimulation effects for all patients. Models derived from pre-stimulation data consistently outperformed the null model when predicting neurostimulation effects on both tasks’ performance. Notably, we could predict behavioural declines just as well as improvements. In conclusion, inter-patient variation in online responses to neurostimulation is, to some extent, systematic and predictable. Since declines in performance were just as predictable as improvements, the behavioural effects of neurostimulation in patients with anomia are unlikely to be driven by placebo effects. However, the online effect of the intervention appears to be as likely to interfere with task performance as to improve it.

## 1. Introduction

Anomia is perhaps the most common symptom of acquired language impairment [1]. Anomia primarily presents as a difficulty with naming common objects. Treatments for anomia typically depend on massed practice and require high doses to work [2]. To really achieve any benefit, patients and therapists need to invest significant time and effort. To try to alleviate that effort, research interest has increasingly turned to potential accelerants of treatment for aphasia, including drugs [3] and neurostimulation [4]. One promising example of the latter type of intervention is transcranial Direct Current Stimulations (tDCS), which is both relatively inexpensive and tolerable by most patients [5].

A typical tDCS setup involves two electrodes: an active electrode and a return electrode. The active electrode is placed on the scalp over the target brain region. The return electrode is placed elsewhere, either on the head (e.g., over the contralateral supraorbital area) or on a region away from the head, such as the contralateral shoulder. The tDCS electrodes interact to create an electrical field that alters cell membrane potentials. In turn, this modulates the likelihood of neuronal firing in and potentially around/beyond the target brain region [6]. Exactly what kind of modulation it makes is still a matter of debate. In broad terms, cathodal tDCS is thought to decrease the excitability of neurons in the target region, while anodal tDCS is thought to increase their excitability. However, this dichotomy is derived from studies stimulating the motor cortices [7], leaving the precise effects on other brain regions largely unknown [8]. And, even when stimulating the motor cortices, those precise effects depend on the details of the set-up (e.g., the size of the electrodes, the distance between them, and the intensity of the stimulation) [9].

Nevertheless, there is increasing evidence that tDCS can be used to modulate language task performance, both in the neurologically normal [5,10,11] and the damaged [4,6,9,12] brain. In the study of anomia, one natural stimulation site is the left inferior frontal gyrus (LIFG). Activity in this region (which includes Broca’s area) has a long association with semantic language [13], and the same activity is known to correlate with naming performance in stroke patients with aphasia [14,15,16,17]. Consistent with these findings, anodal tDCS was shown to enhance stroke patients’ naming accuracy when applied over the LIFG [18]. However, the evidence here is inconsistent because other studies either failed to find the effect or found it but only with cathodal tDCS (e.g., [19]).

One reason for this inconsistency might be that there is no real effect. If this is true, the modest effects reported [4,12] might be artefacts of within-subject noise in those experiments. Alternatively, the effects might be real but also small enough that they are hard to distinguish because of the between-subject noise. In either case, one might justifiably doubt the effects’ clinical relevance. However, even in studies that fail to find significant group effects, at least some individual patients often respond well [20]. The primary aim of this study was to test the claim that those responses are really artefacts of noise.

Following the model of our own recent work in the study of therapy effects [21], we test this by trying to predict individual responses with models trained only on other patients’ data. If we can find enough consistency to make those predictions, the implication is that the individual variance cannot be (wholly) driven by noise. From this perspective, apparently modest group-level effects might belie more complex individual-level effects. Some subset of the participants might be more likely to respond, perhaps much more significantly, than the group-level literature suggests [21]. To the extent that these online effects predict longer-term gains [18], we could then stratify patients for tDCS-enhanced treatment by identifying those who are most likely to benefit.

Our focus in what follows is on a study designed to measure online neurostimulation effects on naming performance, using a size judgement task as a control [22]. We ask whether neurostimulation responses in either task are predictable. Our secondary hypothesis concerns the handling of anomic patients whose lesions damaged the LIFG. We assumed that patients would not respond well to neurostimulation over the damaged cortex, so we needed an alternative stimulation site for these patients. Following prior evidence of right homologue adaptation during recovery in patients with LIFG damage (e.g., [23]), we chose to stimulate the right homologue of LIFG (i.e., RIFG) in these patients.

Anodal stimulation was applied at each site (i.e., LIFG/RIFG), following the logic of the Bimodal Balance Recovery (BBR) model [24]. The BBR was initially advanced as a model of recovery from post-stroke hemiparesis, building on prior findings suggesting that the bilateral motor cortices exhibit mutual inhibition [25]. If this is true, one might naturally seek to enhance ipsilateral motor function by using neurostimulation to inhibit contralateral motor activation. This is sometimes called ‘inter-hemispheric rebalancing’ [26]. The BRR model posits an exception to this rule whenever the ipsilateral motor cortex suffered extensive damage. In this case, the theory is that the damage will extinguish or severely reduce the ‘structural reserve’ available to make use of inter-hemispheric rebalancing. So, rather than inhibiting the contralateral hemisphere, the BBR model contends that we might best promote recovery by recruiting (activating) those contralateral homologues [27].

Driven by this model, our secondary hypothesis was that excitatory neurostimulation over RIFG in patients with extensive LIFG damage would yield responses consistent with those for LIFG neurostimulation in patients whose lesions spared the LIFG. We test the hypothesis by training models with data from patients stimulated at one site and using them to predict neurostimulation responses for patients stimulated at the other site. Reasonable predictions are evidence that the BRR model might be applicable to recovery from post-stroke aphasia.

## 2. Methods

### 2.1. The Neurostimulation Experiment

#### 2.1.1. Participants

The study included 36 participants, all of whom survived a first-time left-hemisphere stroke at least one year before entering the study, had normal hearing, normal or corrected-to-normal visual acuity, and no previous history of neurological or psychiatric disease, as well as no contraindications to MRI scanning. Inclusion criteria were as follows: (i) anomia as determined by the naming subtest of the Comprehensive Aphasia Test [28]; (ii) good single-word comprehension as assessed by the spoken words comprehension subtest of the Comprehensive Aphasia Test; (iii) relatively spared ability to repeat single monosyllabic words from the Psycholinguistic Assessments of Language Processing in Aphasia [29]; (iv) absence of speech apraxia as determined by the Apraxia Battery for Adults [30]. Informed consent was obtained from all participants prior to participation under approval from the Central London Research Ethics Committee, UK.

All participants’ lesions were assessed by JC as either sparing or damaging the LIFG. Participants who suffered extensive damage to LIFG were assigned to the Broca’s area damaged (BD) group and stimulated over RIFG. Otherwise, patients were assigned to the Broca’s area preserved (BP) group and stimulated over LIFG. There were 18 participants in each group.

#### 2.1.2. Experimental Design

Participants completed two simultaneous fMRI/tDCS sessions, one with anodal stimulation and the other with sham stimulation, separated by a one-week wash-out period. Sessions were counter-balanced across subjects. In each session, participants completed both a picture naming task and a size judgement task: stimulation responses were measured as their accuracies or reaction times on each task during anodal stimulation minus accuracies/reaction times on the same task during sham stimulation. Each task also manipulated both visual object information (by adding noise to the images) and auditory object information (with phonemic cues), but our stimulation response variables averaged across these conditions.

#### 2.1.3. Behavioural Tasks

The naming task required participants to name each presented object as quickly and as accurately as possible. The judgement task required them to decide whether the object would fit inside a microwave oven (yes/no verbal responses), the aim being to study lexical retrieval in naming while controlling for object identification, decision-making, and a vocal speech response. This experimental design and these stimuli are described in detail in [22].

Each object picture was a black-and-white line drawing of a concrete object. A trial started with a 1 s presentation of a fixation cross, followed by simultaneous appearance of the object picture with the auditory cues (squiggly lines/geometric shapes superimposed on the images and auditory stimuli corresponding to the first phoneme of an object name or its vocoded version). Pictures were displayed for 2.5 s, followed by a blank screen for 0.35 s. The order of experimental conditions was pseudo-randomized within a block (i.e., avoiding more than three trials of the same condition in a row), and objects were counterbalanced across participants to control for psycholinguistic features like frequency, concreteness, imageability, and initial phoneme.

Stimulus presentation and behavioural data acquisition were controlled using Matlab. Overt spoken responses were recorded online using a dual-channel, noise-cancelling fibre optical microphone system (FOMRI III; http://www.optoacoustics.com). These recordings were reviewed offline to calculate speech accuracy scores (% correct) for each participant. Auditory cues were delivered via MR-compatible headphones (MR Confon, Magdeburg, Germany; www.mr-confon.de (accessed on 20 December 2023)). Participants completed a short training period before entering the scanner for each session, both to familiarise themselves with the task and to practice speaking softly, thus minimising motion in the scanner. Stimuli used during the training were not used during the neurostimulation session. A total of 480 target words were selected from the IPNP database [31] (n = 220) and from the MRC Psycholinguistic Database [32] (n = 260). Object names were monosyllabic words with a consonant–vowel–consonant (CVC) phonological structure.

Response accuracy in the judgement task (i.e., deciding whether an object would fit inside a microwave oven) was defined relative to a normative standard derived from a separate experiment run using Amazon’s web-based Mechanical Turk platform (MTurk; see [33]). A total of 32 adult participants viewed all the 480 stimuli that we used in the main experiment (without visual or auditory cues) and used yes/no button press responses to indicate whether each object would fit in a microwave oven. Moreover, 62/480 items did not reach our threshold of 70% agreement in these responses, so they were excluded from the main analysis. Judgement and naming reaction times were timed from stimulus onset until the patient responded. Stimulation responses for both tasks were calculated as the naming accuracy/reaction time during anodal stimulation minus the naming accuracy/reaction time during sham stimulation.

#### 2.1.4. Neurostimulation with Concurrent fMRI

The current analysis makes no reference to the fMRI data that were collected concurrently with the application of neurostimulation. Nevertheless, the tDCS was delivered during an fMRI experiment, so we describe the key elements of that procedure here.

Neurostimulation was delivered using an MR-compatible stimulation system (neuroConn; https://www.neurocaregroup.com/dc_stimulator_mr.html (accessed on 20 December 2023)) via a pair of MR-compatible leads and rectangular rubber electrodes (5 × 7 cm), allowing for a current density of 0.057 mA/cm^2^ (cf. [11]). For those participants identified (by JC, from structural MRI images) as ‘LIFG intact’, the anode was placed over the LIFC (equivalent to position FC5 in 10–20 EEG nomenclature), and the cathode was placed over the contralateral frontopolar cortex (FP2). For participants identified as ‘LIFG damaged’, these stimulation sites were mirrored with the anode placed over the right hemisphere IFG (RIFG). Both electrodes and the sites on the scalp where the electrodes were placed were covered with EEG conductive paste to ensure a flush and comfortable fit between the electrode surface and the scalp. Electrodes were secured to the head using 3M Coban elastic wrap bandage and placed in adherence with the manufacturer’s MR safety guidelines. Care was taken in connecting the leads backward along the centre of the scanner bore to minimise the possibility of radio frequency-induced heating and to ensure that any gradient switching-induced AC currents were well below the level that might cause stimulation. The stimulator was placed outside the Faraday cage of the scanner, and the stimulating current was fed to the participant through two stages of radio frequency filtration to prevent interference from being picked up by the scanner.

A scanner pulse triggered the onset of the stimulation at a given slice in the acquisition sequence. The current was increased slowly during the first 15 s to the desired stimulation threshold (2 mA), termed the ramp-up phase. A constant direct current (2 mA) was delivered for 20 min. At the end of the stimulation period, the current was decreased to 0 mA over 1 s (ramp-down). For sham stimulation, the ramp-up phase was followed by 15 s of 2 mA stimulation, which was immediately followed by a 1 s ramp-down phase. This active sham protocol makes for a more efficient double-blinding process: both stimulation and sham protocols produced sensations of comparable quality (a mild tingling, typically under the electrode placed over the contralateral orbital/frontopolar edge). Participants habituated to the sensation quickly and reported minimal discomfort with no adverse sensations during both anodal and sham tDCS stimulation runs. The position of the anode and cathode electrodes for each subject was recorded and reproduced across scanning sessions. Full details of the tDCS-fMRI procedure validated both quantitatively and qualitatively (in the same MRI scanner used to collect the present data) can be found in Nardo et al. [34].

#### 2.1.5. Structural MRI Acquisition and Analysis

Structural MRI was acquired during the same scanning sessions as the simultaneous tDCS and fMRI. Whole-brain structural imaging was performed on a 3T Siemens TIM-Trio system (Siemens, Erlangen, Germany) at the Wellcome Centre for Human Neuroimaging. T2*-weighted echo-planar images (EPI) with BOLD contrast were acquired using a 12-channel head coil. Structural MRI data were processed into binary lesion images using the Automatic Lesion Identification toolbox [35], running under SPM 12 in Matlab. We employed the (120,231) left-hemisphere voxels from the “fuzzy” lesion images produced by the toolbox to represent patients’ lesions. Voxel values in these regions range from 0 to 1, with higher values indicating greater evidence that the voxel is damaged, and evidence is derived by comparing tissue intensity in each voxel to intensities from a population of neurologically normal controls.

### 2.2. Data Analysis

#### 2.2.1. Data

Our key interest here was in whether behavioural neurostimulation responses might be predictable at the individual level. Neurostimulation responses are differences in average performance in both naming and judgement tasks during either sham or anodal stimulation. We consider two types of difference—in accuracies and reaction times—for each of the two tasks, leading to four dependent variables. We attempt to predict these four dependent variables in four separate analyses.

Our independent variables, or predictors, were defined based on our own past work attempting to predict individual responses to neurorehabilitation [36]. These included the following: (a) clinical data, (b) lesion data, and (c) behavioural data. All were acquired at baseline before the neurostimulation part of the experiment began.

The clinical data included the following: age at stroke onset, sex assigned at birth, time post-stroke, pre- and post-stroke handedness, years of formal schooling, and total lesion volume. The lesion data consisted of lesion probability values from a total of 120,213 2 mm^3^ voxels from the left hemisphere of the Montreal Neurological Institute brain. The behavioural variables were derived from a comprehensive battery of language and non-language tests administered to assess participants’ language and cognitive abilities. The tests included the following: the (full) Comprehensive Aphasia Test [28], the PALPA 8 and 9 [29], the Boston Naming Test [37], the Pyramids and Palm Trees test [38], the Cattel Culture Fair IQ Test (Scale 2, Form A) [39], the Rey–Osterrieth Complex Figure Test [40], digit-span tasks from the Wechsler Adult Intelligence Scale [41], the Delis–Kaplan Executive Function System test [42], the Hopkins Verbal Learning Test [43], and the Children’s Sustained Attention to Response Task [44]. A complete list of all the 96 variables derived from these tests is included in Supplementary Materials. A very small number of these behavioural variables was missing for some patients (0.69% of the behavioural data values), and we used mean imputation to fill in these missing values.

As mentioned previously, we were interested in whether the neurostimulation responses (or dependent variables) might be predictable, given these independent variables. But we were also interested in which data types (clinical, behavioural, or lesion) were more predictive and in what combination. So, we trained and tested machine learning models from each data type separately and from every combination of those data types. With three data types to consider, this gave us a total of 7 combinations of predictors to try for each of 4 dependent variables. So, the whole analysis consists of training and testing a total of 28 different models.

#### 2.2.2. Associating Predictors to Responses with Partial Least Squares Regression

Given the very large number of predictors relative to the sample size (n = 36), we employed Partial Least Squares (PLS) regression to reduce the predictors’ dimensionality efficiently. PLS regression employs a data dimensionality reduction procedure analogous to Principal Components Analysis (PCA). In PCA regression, one would first derive latent variables from the independent variables via PCA, then select some subset of those variables (typically the first N components), and then perform a standard linear regression. By contrast, where PCA would derive latent variables that explain as much of the variance of the independent variables as possible, PLS regression derives them to explain dependent variable variance (neurostimulation responses, in our analysis). PLS regression should, thus, be more efficient than PCA regression and, perhaps, therefore, more robust to high-dimensional data.

#### 2.2.3. Model Assessment and Model Comparison

Predictive performance was assessed with 100 times 10-fold cross-validation. First, we divide the sample into 10 groups. Then, we train a PLS regression model with data from participants in 9 of those groups and use the trained model to predict responses for the participants in the 10th group. The only hyperparameter that requires tuning in these models is the number of components to employ for the data dimensionality reduction step. We set this number in a nested manner, using an inner loop of 10-fold cross-validation that operates only on the training data for each fold of the main cross-validation loop. We repeat this procedure 10 times so that every group is the test group exactly once. And then we repeat that whole procedure 100 times, with 100 different partitions of the sample into 10 groups. Resultant measures of (mean squared) prediction error are averages across these folds and repetitions. Notably, all folds of all cross-validation analyses (i.e., the ways in which the original sample is divided into 10 groups) are kept constant across all model configurations.

We assessed our models in relative terms, by comparing them each to a baseline model. The baseline model assumes that individual variance in neurostimulation responses is not predictable, so it simply predicts the mean neurostimulation response for all participants. When our data-driven models outperform this baseline model (have smaller prediction errors), we can conclude that individual treatment responses are predictable, at least to some extent. We use the traditional, paired, non-parametric Wilcoxon signed rank test to compare MSEs across models and further threshold those statistics with a paired permutation test. The test construes the two vectors to be compared as having labels reflecting the models used to generate them. The null hypothesis is that those labels are arbitrary because the models’ performances differ only by chance. By shuffling those labels, we can, therefore, create a null distribution of paired test statistics. If the real comparison between the real sets of prediction errors yields statistics that are extreme relative to the null distribution (*p* < 0.05), we conclude that the performance difference between the models is significant.

#### 2.2.4. Model Interpretation

PLS regression models can be interpreted by examining the weights of each of their components on each of the original variables. However, this approach can be challenging when there are multiple components to consider and because the sign of each component is arbitrary: i.e., positive weights on a given component do not necessarily imply a positive relationship between the highly weighted independent variables and the dependent variable(s). We circumvent these issues with ‘data perturbation’.

Having first trained a PLS model on the original data, we then used that trained model to make further predictions after randomly shuffling the predictors. Having repeated this procedure 1000 times, we will have 1000 sets of predictions for 1000 sets of permuted predictors. We calculate the influence that each predictor value has on the predictions as the (Pearson’s) correlation between the two. If the predicted value increases when a predictor value increases (and vice versa), that predictor will obtain a positive weight. By contrast, if the predicted value decreases when the predictor value increases (and vice versa), that predictor will obtain a negative weight. To confirm the stability of the weights assigned using this data perturbation procedure, we ran the whole process 10 times (i.e., for a total of 10,000 random perturbations). Predictor weights assigned in any 1000-iteration block were very strongly correlated with weights assigned in other blocks (all r ≥ 0.99).

When using lesion data, we have >100,000 voxel values to consider and, therefore, >100,000 weights on those voxels. We report these weights after first aggregating them within series of region masks drawn from publicly available atlases [45,46,47,48]. This region-level encoding allows us to summarise the voxel-level coefficients at the level of brain regions and white matter tracts.

## 3. Results

### 3.1. Patient Characteristics

**Figure 1:**
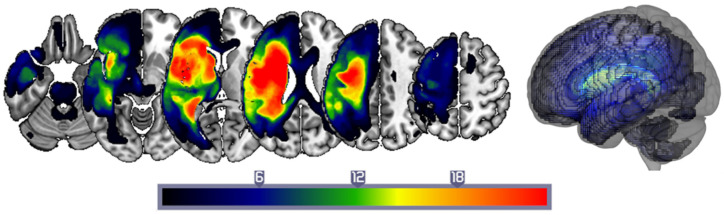
**Lesion frequency plot** for the sample of 36 patients.

Our sample of 36 stroke patients included 26 men and 10 women. The mean age was 59.1 years (±12.5 years), the mean time post-stroke at assessment was 10.4 yrs (±8.0 years), and the patients’ mean years of schooling was 13.8 years (±2.4 years). The sample was divided into two sub-groups, depending on whether their lesions preserved or partially preserved the left inferior frontal gyrus (Broca’s area preserved: BP or Broca’s area destroyed: BD). The two groups were not significantly different in age at stroke onset, time post-stroke at assessment, or years of schooling (all *p* > 0.1). The mean lesion size across the whole sample was 125.0 cm^3^ (±106.4 cm^3^), with patients in the BD group suffering significantly larger lesions (183.3 cm^3^ ±115.1 cm^3^) than the BP group (66.7 cm^3^ ±106.4 cm^3^): Z = 3.33, *p* < 0.001. See Figure 1 for a lesion frequency plot.

### 3.2. In-Scanner Behaviour and Stimulation Responses

Table 1 reports participants’ in-scanner performance under both sham and anodal stimulation. Under sham stimulation, the BP group was significantly more accurate than the BD group in naming (Z = 2.48, *p* = 0.013) but not judgement (Z = 1.43, *p* = 0.150). The BP group was also numerically faster than the BD group in the judgement task, though this did not reach significance (Z = 1.91, *p* = 0.057), and there was also no significant difference in reaction times for the naming task (Z = 0.21, *p* = 0.837). Under anodal stimulation, the results were similar in that the BP group was more accurate in naming than the BD group (Z = 2.86, *p* = 0.004), and there were no other significant differences between the groups (all *p* > 0.3).

**Figure 2:**
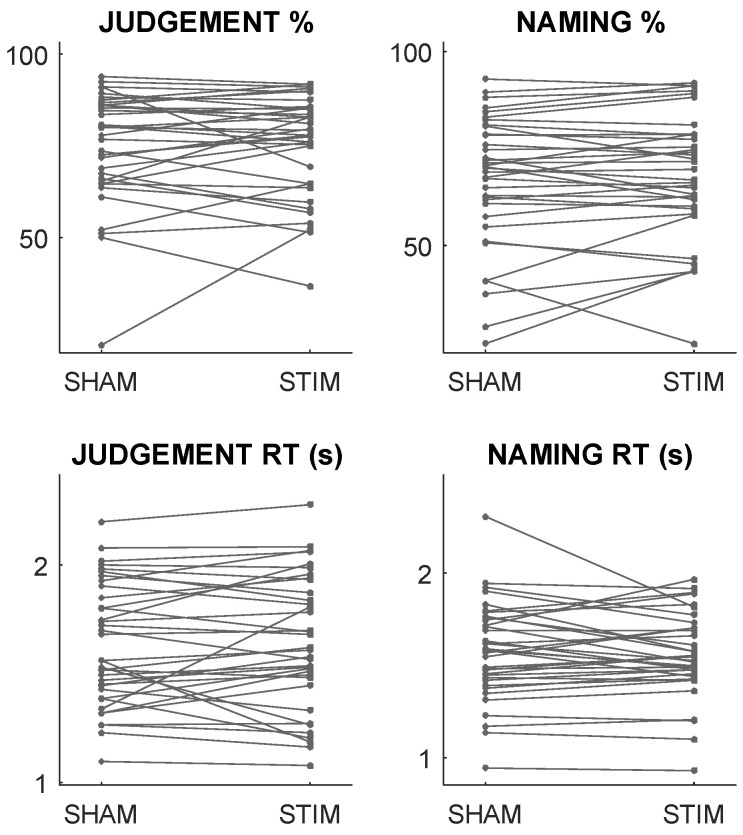
**Stimulation responses.** Mean accuracies (**top**) and reaction times (**bottom**) in sham vs. anodal stimulation and in the judgement (**left**) and naming (**right**) tasks. The numbers of patients whose stimulation responses were positive (/negative) in each case were as follows: judgement accuracies: 15 (21); naming accuracies: 20 (16); judgement reaction times (measured in seconds): 20 (16); naming reaction times: 19 (17).

Crucially, there were no significant effects of anodal neurostimulation (compared to sham stimulation), either on accuracies or on reaction times in either task, either across the group as a whole or in either of the two sub-groups: all *p* > 0.2. This is despite evident variance at the individual level in all four independent variables: i.e., the stimulation appears to significantly alter some patients’ accuracies and/or reaction times, even though there is no consistent, group-level effect. We hypothesised the null effects at the group level might belie a more systematic structure in the patients’ individual responses to neurostimulation, which are illustrated in Figure 2. We reasoned that if the changes themselves are predictable, these individual-level effects cannot be dismissed as artefacts of noise.

### 3.3. Predictive Performance

With three data types to consider (clinical, behavioural, and lesion data), we employed a total of eight model configurations: i.e., seven combinations of predictor data types, plus an eight combination for the null model, which does not employ any predictor data (because it simply predicts the mean stimulation response of the patients in its training sample). Table 2 records the results, highlighting the best models for stimulation responses (accuracies and reaction times) in the judgement and naming tasks, respectively.

All our dependent variables—stimulation responses for accuracies and reaction times in the naming and judgement tasks—appeared to be predictable. Each could be predicted by some combination of pre-stimulation data at better (lower) error rates than those associated with the null model. Lesion data were a component of the best models’ predictors for one-third of the dependent variables. The exception was for predicting stimulation effects on reaction times in the judgement task, where the best model included clinical and behavioural data only (see Table 2); however, lesions alone were also able to make better-than-null-model predictions for this variable.

The best models for each response variable were also all significantly better (after FWE correction) than the next best, implying that their advantages, though numerically small, were highly consistent across cross-validation folds. The best models were also all very significantly better than the null model: all FWE *p* < 0.001. These results imply that the small advantages enjoyed by these models are very consistent across the 100 repetitions of the cross-validation process. Since all stimulation response variables were standardised prior to running the predictive analyses, their MSEs are also comparable. Stimulation effects on naming accuracy appeared more predictable than stimulation effects on naming reaction times, whereas this order was reversed for the judgement task.

Finally, no model relied simply on predicting improvements (increasing accuracies or decreasing reaction times) consequent to neurostimulation. If any model only predicts change in one direction, it follows that the predictions for those patients whose performance changed in the other direction should be less accurate. To test this, we employed a Bayesian analysis of variance [49], measuring whether the direction of empirical change (stimulation responses greater versus lesser than sham responses) mediated the MSEs of each of our models’ mean predictions. In fact, there was strong evidence *against* a mediating effect for all dependent variables: BF = 0.04 (judgement accuracy); 0.04 (judgement reaction times); 0.03 (naming accuracy); and 0.08 (naming reaction times). As in past work [50], we interpret these results as evidence of systematicity/predictability in both the improvements and the declines in performance, here associated with neurostimulation.

### 3.4. Examining the Role of Stimulation Site

One feature of the experimental design deserves consideration: half of the patients in this sample were stimulated in the LIFG, whereas the other half, with no or minimal preservation of LIFG after stroke, were stimulated in the RIFG. If responses to stimulation at each site are different, our models might have simply discovered this feature of the experimental design, confounding our results. There were no significant differences in the stimulation responses for each group in either the judgement task (Wilcoxon rank sum test; accuracies: z = 0.98, *p* = 0.33; reaction times: z = 1.12, *p* = 0.26) or the naming task (accuracies: z = 1.60, *p* = 0.10; reaction times: z = 0.97, *p* = 0.33). However, this maintains the possibility that there is some subtler interaction, for example, with the lesion site, which drives the whole-sample predictive results.

One way to test this would be to repeat the analyses described in the last section for each sub-group separately. However, this test is confounded by the significant reduction in the sample sizes of each sub-group relative to the whole group, which is already smaller than might be preferred for predictive modelling. Instead, we compared the performances of our best models for each task when predicting between stimulation sites to their performance when predicting across stimulation sites.

If the stimulation site is a key driver of the original whole-sample results, it should be particularly important to train our models with some examples of patients who were stimulated at LIFG and some examples of patients who were stimulated at RIFG. It follows that predictions for each sub-group based on training only with the other (‘between-group splits’) should be less accurate than predictions derived based on training with exemplars from both sub-groups (‘across-group splits’). We tested this by running each of our four best PLS regression models (one for each dependent variable) through split-half cross-validation with the between-group split and comparing its MSE to a distribution of MSEs obtained for the same model over 1000 cross-validation runs with across-group splits. To the extent that our results for each dependent variable are confounded by the stimulation site, we expected more across-group MSEs to be smaller than the between-group MSE for that variable.

The proportions of across-group MSEs that were, in fact, smaller than each between-group MSE were as follows: 84% (judgement accuracies), 15% (naming accuracies), 61% (judgement reaction times), and 36% (naming reaction times).

Quite how high these proportions would need to be to demonstrate a stimulation-site confound remains an open question. A threshold of >95% seems consistent with the standard definition of significance but might be considered too conservative here. Instead, we interpret these proportions as convincing evidence that the stimulation site is not driving our results for reaction times in both tasks and for accuracy in the naming task because they imply that the between-group MSE was at least within the inter-quartile range of those across-group MSEs. Neurostimulation-induced changes in judgement accuracy, however, were outside that range (84%), suggesting that stimulation-site confounds might be more relevant in this case.

### 3.5. Interpreting the Best Models

**Figure 3:**
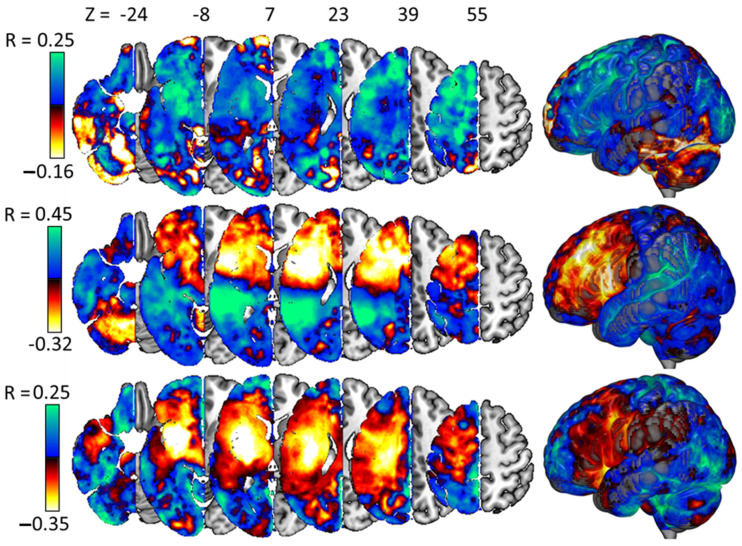
Weights on the lesion voxels in our best models for stimulation responses for accuracies in the judgement task (**top**) and naming task (**middle**) and for reaction times in the naming task (**bottom**). Note that no brain image is shown for the model predicting stimulation responses on reaction times in the judgement task because lesion data played no part in the best model for that variable.

Each of the four best PLS regression models assigns non-zero weights to all its predictors, and in this sense, none of the predictors are strictly redundant in any model. Nevertheless, while we report all model weights, either in Figure 3 below (for lesion-related weights) or in the Supplementary Materials (for non-lesion weights), for the sake of parsimony, we concentrate only on the most important variables here, i.e., those assigned the weights of the largest magnitude.

In those models that included lesion data, the strongest weights, implying that more damage drives less improvement/more decline in performance after neurostimulation, were assigned to the inferior temporal cortex (judgement accuracy), the putamen (naming accuracy), and the superior longitudinal fasciculus (SLF) (naming reaction times). Note that in the first two cases, these weights were negative because higher accuracies imply better performance, but in the third case (naming reaction times), the weight was positive, implying a greater tendency to lengthen reaction times with more damage to the SLF. In these models, the strongest weights implying the opposite relationship, with more lesion damage predicting worse responses to neurostimulation, were found in the cuneus (judgement accuracy: 0.53) and the cingulum (naming accuracy: 0.50 and naming reaction times: 0.50).

In those models that included behavioural data, the strongest weights predicting greater performance improvement with better pre-stimulation performance were on scores in the Rey immediate copy task (0.04; judgement accuracy), successful self-corrections in confrontation naming (0.05; naming accuracy), and the PALPA 9 repetition task (0.10; judgement reaction times). In the same models, the strongest weights implying the opposite relationship, predicting worse responses to neurostimulation with better pre-treatment performance, were scores on the CAT verbal fluency task (0.06; judgement accuracies), the CAT summary score for all auditory comprehension tasks (0.09; naming accuracy), and the recognition subtest of the Hopkins Verbal Learning Test (0.23; judgement reaction times).

Finally, in those (two) models that employed clinical data, the strongest weights were on time post-stroke when predicting naming accuracies (−0.06), implying that patients assessed sooner after stroke responded better to the neurostimulation. When predicting judgement reaction times, the strongest weight was on total lesion volume (0.14), suggesting that patients with larger lesions tended to have longer reaction times during anodal rather than sham stimulation.

## 4. Discussion

### 4.1. Inter-Individual Variance in Neurostimulation Responses Is Predictable

TDCS has attracted growing attention as a potential accelerant for the rehabilitation of aphasia [12], and evidence suggests that the immediate, online impact of tDCS might predict those longer-term rehabilitation effects [4]. However, the real impact of tDCS is still somewhat unclear because, while many studies report significant facilitation of language via tDCS (e.g., [4,10,19,51]), these effects appear modest and inconsistent [6,9,12].

One potential explanation for this inconsistency follows from a recent study of the effects of tDCS on verbal fluency in the healthy (young) adult brain [20], in which the authors reported a ‘paradoxical’ decline in performance rather than the expected improvement. If these interference effects can also occur in the damaged brain, modest or null neurostimulation effects at the group level might belie a more complex dichotomy of improvements and declines at the individual level. Certainly, a visual inspection of our own patients’ neurostimulation responses is consistent with this notion because, for some patients, tDCS improves performance, while in others, it declines (see Figure 2).

Notably, this is also the expected pattern if neurostimulation responses were driven entirely by (measurement and/or stimulation-induced) noise. However, noise-induced changes should not be predictable, and many of the models that we tested made significantly better predictions than those of a baseline model, in which each patient’s prediction was simply the mean stimulation response of the group in each task (see Table 1). We also found strong [52] evidence against a mediating effect of the direction of change on the predictability of change: i.e., both directions of change were (approximately) equally predictable. Since neurostimulation responses were significantly predictable, and both directions of response appeared equally predictable, it follows that neither direction of response is an artefact of noise. We hope that this result will encourage future reports of these studies to distinguish participants whose performance declines with tDCS rather than simply distinguishing ‘responders’ from ‘non-responders’, as is currently common [12].

### 4.2. The BRR Model Is Supported

Notably, it was typically also just as easy to predict stimulation responses for one stimulation site (left inferior frontal gyrus) after training only with patients stimulated at the contralateral homologue (and vice versa) as it was when training with exemplars from both stimulation sites. The implication is that, to some approximation, the functional role of the LIFG in patients whose strokes spared it is comparable to that of the RIFG in patients with severe damage to the LIFG. As far as we know, this is the first evidence that the Bimodal Balance Recovery (BBR) model [24] might apply to recovery from aphasia, as well as motor impairment, after stroke.

### 4.3. Model Weights Are (Often) Plausible in the Context of Prior Research

The weights assigned by each of our four best models were broadly plausible. The emphasis on the inferior temporal cortex (ITC), in the best model for judgement accuracies, is consistent with the role that the ITC is thought to play in object recognition via the ventral visual stream [53,54]. The emphasis on the putamen and supervening white matter in the model for naming accuracies is consistent with that region’s extensive connectivity to left hemisphere language networks [55]. And the emphasis on the superior longitudinal fasciculus in the model for naming reaction times is also consistent with studies relating the preservation of this tract to long-term language outcomes after stroke [56,57]. Notably, lesion damage in each of the regions that were positively associated with neurostimulation responses (i.e., more damage = better response) was significantly anti-correlated with lesion damage in those regions where the damage had the more intuitive association with neurostimulation responses (i.e., more damage = poorer response). This suggests that these models’ more counter-intuitive weights (more damage = better response) might reflect the contingent distribution of the lesions suffered by these patients. In other words, regions where more damage appears to improve responses to neurostimulation might best be explained as regions where damage predicts the preservation of other regions required to benefit from the intervention.

Weights on behavioural variables were more difficult to interpret. The weight on the immediate-copy condition from the Rey Complex Figure Task, in the model for judgement accuracies, is sensible in that it implies that better recovered or more preserved executive functioning skills, visuospatial abilities, and/or working memory [58] after stroke, might encourage better responses to neurostimulation in the size judgement task. The weight on self-corrections of errors in confrontation naming in the model for naming accuracies is plausible in that it links better preserved/recovered error monitoring in naming to improvements in naming capacity consequent to neurostimulation.

However, the emphasis on scores in the PALPA9 word repetition task in the model for judgement reaction times (better scores = faster responses) is difficult to explain. And in all models, counter-intuitive weights (better pre-stimulation performance predicts worse stimulation responses) were stronger than intuitive weights. This perhaps suggests an element of regression to the mean, with patients suffering greater impairment more likely to respond better to neurostimulation and vice versa.

### 4.4. Limitations

However, given the relatively small sample size (n = 36) compared to the number of predictors that we considered (>100,000, when lesion data are included), we would caution against strong interpretations of these weights. Indeed, sample-size limitations impose quite strict limits on our confidence in all our results.

Our modelling approach offers a principled and (largely) unbiased way to measure whether stimulation responses are predictable in principle. This method is also, demonstrably, robust to the high dimensionality of the data that we considered. But with the sample we have, it might be difficult or impossible to identify the ‘best’ (or perhaps even ‘right’) models to predict neurostimulation responses. We assume that these models will include only a subset of the independent variables that we considered, and there is no guarantee that the weights on those variables will be consistent with what we see here. Attempts to identify that subset via feature selection are highly susceptible to overfitting in feature selection space [21]. In theory, statistical significance tests on model weights might be a viable alternative approach to revealing a subset of predictors whose influence is expected to be stable [59]. However, results like this might give the (fallacious) impression that those weights can be interpreted in isolation, which is why we avoided it here. Another attractive, if heuristic, alternative would be to explore sparse variants of Partial Least Squares regression (e.g., [60]). This is a focus of ongoing work. But though we can mitigate the limits of small-sample inference with careful methodology, we cannot hope to completely extinguish those limits. And since these therapy studies are extremely resource intensive, small-sample limits are likely to remain significant in all or most work like this, at least in the near future.

Similarly, our current results are limited because our sample is not representative of the population of patients with anomic stroke. First, we only considered patients in the chronic phase post-stroke, so our results cannot speak to neurostimulation effects in patients whose strokes were more recent. Second, we selected patients whose lesions were restricted to the left hemisphere of the brain, so we cannot know if our models would generalise to patients with right hemisphere or bilateral lesions. And third, our sample was selected to clarify the effect of neurostimulation on the contralateral and ipsilateral IFG. Therefore, we had to select a sample of participants whose lesion distribution might well depart from that of the wider population of stroke survivors with anomia. Moreover, lesion site differences confound any analysis of the detailed differences between responses to neurostimulation over either the LIFG or the RIFG. This is because patients were only stimulated over the LIFG if that region was preserved and because the LIFG is known to be an important node in the neuroanatomical network that is thought to implement naming skills.

## 5. Conclusions

Nevertheless, and despite their limitations, our results suggest that individual responses to neurostimulation in both judgement and naming tasks are at least somewhat systematic and predictable. Stimulation-induced declines in performance appeared to be just as predictable as stimulation-induced improvements, encouraging a greater focus on ‘decliners’ in future neurostimulation studies, in addition to ‘responders’ and (possibly) ‘non-responders’. And the apparent ease with which we could predict stimulation responses with the between-site split adds confidence that responses to stimulation at each site were consistent or at least consistently related to patients’ behavioural impairments and lesions. We hope that these results will encourage a more personalised approach to the application of neurostimulation in post-stroke aphasia, maximising its potential to improve the efficiency of therapies for post-stroke aphasia.

## Figures and Tables

**Table 1:** **In-scanner performance.** Accuracies and reaction times (mean and standard deviation) for the whole sample and both sub-groups in the naming and judgement tasks, respectively, under both sham and anodal stimulation. Bold, underlined font highlights where the BP group was significantly more accurate than the BD group in naming under both tDCS conditions (sham: Z = 2.48, *p* = 0.013; anodal: Z = 2.86, *p* = 0.004).

		ALL	BP	BD
SHAM	Judgement %	74.9 (±15.2)	78.2 (±13.7)	71.6 (±16.4)
	Judgement RT (s)	1.63 (±0.28)	1.54 (±0.31)	1.72 (±0.24)
	Naming %	66.8 (±16.9)	**72.2 (±16.6)**	**61.3 (±15.9)**
	Naming RT (s)	1.57 (±0.26)	1.57 (±0.23)	1.57 (±0.29)
ANODAL	Judgement %	75.5 (±13.8)	77.0 (±13.3)	73.9 (±14.6)
	Judgement RT (s)	1.64 (±0.31)	1.59 (±0.34)	1.70 (±0.27)
	Naming %	67.8 (±16.0)	**74.7 (±14.2)**	**60.8 (±14.8)**
	Naming RT (s)	1.55 (±0.23)	1.58 (±0.24)	1.52 (±0.22)

**Table 2:** **Model predictive performances.** Median and inter-quartile ranges of Mean Squared Errors (MSE) achieved by models driven by different combinations of data across 1000 repetitions of a 10-fold cross-validation process. The bold, underlined figures in each column represent best-case performance when predicting that type of neurostimulation response. Folds are kept consistent across models. The best-performing accuracy models, with the lowest median MSE, employed behavioural and lesion variables for the judgement task and clinical, behavioural, and lesion variables for the naming task. DEM—demographics; BEH—behavioural data; LES—lesion data. Notably, all stimulus–response variables were standardised here prior to learning, so MSEs are comparable across them.

DATA	JUDGEMENT %	NAMING %	JUDGEMENT RT	NAMING RT
NULL	1.034/0.033	1.028/0.027	1.033/0.033	1.032/0.030
DEM	1.213/0.075	0.984/0.057	1.315/0.090	1.379/0.115
BEH	1.146/0.081	0.838/0.091	0.861/0.128	0.980/0.093
LES	0.846/0.120	0.780/0.095	0.828/0.073	**0.916/0.080**
DEM + BEH	1.153/0.078	0.775/0.09	**0.723/0.118**	1.020/0.040
DEM + LES	0.848/0.121	0.779/0.094	0.830/0.073	0.917/0.08
BEH + LES	**0.842/0.107**	0.749/0.087	0.819/0.067	0.919/0.08
DEM + BEH + LES	0.845/0.107	**0.748/0.087**	0.821/0.070	0.933/0.066

## Data Availability

The data described in this study are available to accredited researchers from JC upon request. The data are not publicly available due to privacy restrictions in accordance with consent provided by participants.

## Supplementary Materials

The following supporting information can be downloaded at: https://www.mdpi.com/article/10.3390/life14030331/s1, Table S1: Weights on clinical variables where they appear in the best model for a given stimulation response variable; Table S2: Weights on behavioural variables where they appear in the best model for a given stimulation response variable.
