# Supplementary material for: Predicting Online Behavioural Responses to Transcranial Direct Current Stimulation in Stroke Patients with Anomia
Source: Life (Basel). 2024 Mar 1;14(3):331. doi: 10.3390/life14030331 (PMC10971124; doi:10.3390/life14030331)
Supplement: Supplementary file 1 [file life-14-00331-s001.zip › life-2812532-supplementary.pdf]

Supplementary Material:

A. Behavioural variables

In what follows, we list all 96 behavioural variables that are considered in the main analyses.

1. Total score in all CAT comprehension tasks for auditory stimuli
2. Total score in all CAT comprehension tasks for visual stimuli
3. Total score in all CAT auditory repetition tasks
4. Total score in all CAT picture naming tasks
5. CAT semantic memory task
6. Total score in all CAT verbal fluency tasks
7. CAT category fluency task
8. CAT letter fluency task
9. CAT recognition memory task
10. CAT object use gesture task
11. CAT arithmetic task
12. CAT task on the comprehension of spoken words
13. CAT task on the comprehension of written words
14. CAT task on the comprehension of spoken sentences
15. CAT task on the comprehension of written sentences
16. CAT task on the comprehension of spoken paragraphs
17. CAT single-word auditory repetition task
18. Performance when repeating single-syllable words (subset of the above)
19. CAT complex word repetition task
20. CAT non-word repetition task
21. CAT digit span task
22. CAT sentence repetition task
23. CAT object (picture) naming task
24. CAT action (picture) naming task
25. CAT scene (picture) description task
26. Appropriate words used in the scene description task
27. Inappropriate words used in the scene description task
28. CAT word reading task
29. CAT complex word reading task
30. CAT function word reading task
31. CAT non-word reading task
32. CAT written scene (picture) description
33. CAT writing to dictation task
34. PALPA8 non-word repetition task
35. PALPA8 non-word reading task
36. PALPA9 word repetition (High Imageability High Frequency)
37. PALPA9 word repetition (High Imageability Low Frequency)
38. PALPA9 word repetition (Low Imageability High Frequency)
39. PALPA9 word repetition (Low Imageability Low Frequency)
40. PALPA9 non-word repetition task
41. Boston Naming Test (cued and uncued)
42. Boston Naming Test (cued)

43. Boston Naming Test (uncued)
44. Confrontation Naming task (exactly correct responses)
45. Confrontation Naming task (response is a synonym of correct response)
46. Confrontation Naming task (errors self-corrected to correct responses)
47. Confrontation Naming task (multiple word answers containing correct responses)
48. Confrontation Naming task (total correct responses)
49. Confrontation Naming task total semantic errors
50. Confrontation Naming task total phonemic errors
51. Confrontation Naming task total omission errors
52. Confrontation Naming task miscellaneous errors
53. Confrontation Naming task coordinate errors (response is a different item belonging to the same category as the target word).
54. Confrontation Naming task superordinate errors (response is the target word's superordinate category).
55. Confrontation Naming task superordinate errors (response is a more specific word to the target and does not match the picture).
56. Confrontation Naming task associate errors. Response is semantically associated to the target word (noun, verb or adjective) and does not fit into any of the other semantic error categories. This includes sound effects that demonstrate semantic knowledge of the target word.
57. Confrontation Naming task part-whole errors. Response is the correct for a part of the whole picture. Alternatively, a response is correct for the whole picture, rather than the target part.
58. Confrontation Naming task circumlocution errors. Response is a meaningful, multiword description of the target without producing the target word.
59. Confrontation Naming task formal phonemic errors. Response with at least 50% phonemes overlapping with the target word in any position to create a real word.
60. Confrontation Naming task phonemic errors. Response with at least 50% phonemes overlapping with the target word (or an accepted synonym) in any position to create a non-word.
61. Confrontation Naming task partial phonemic errors. Response has at least one phoneme that is related or unrelated to the target
62. Confrontation Naming task neologism errors. Response is a nonword that does not fulfil the above phonemic error criteria.
63. Confrontation Naming task formal omission (incorrect) errors. Response that does not fit into any other category, does not have any semantic relationship to the target. This also includes stereotyped responses.
64. Confrontation Naming task omission (missing) errors. No response is given
65. Confrontation Naming task perseveration errors. Perseverated response repeated from a recent previous target.
66. Confrontation Naming task mixed errors. Response contains a mix of semantic and phonemic errors
67. Confrontation Naming task miscellaneous (unrelated) errors. Response is a real word, but semantically or phonologically unrelated to the target word.
68. Confrontation Naming task self-wrong errors. Response is initially correct but changed to an incorrect response
69. Confrontation Naming task visual errors. Response that is visually similar to the target but is not semantically related.
70. Pyramids and Palm Trees Test
71. Cattel Culture Fair IQ Test, Scale 2, Form A

72. RBM
73. Rey-Osterrieth Complex Figure Test, immediate copy condition
74. Rey-Osterrieth Complex Figure Test, 3-minute delay copy condition
75. Rey-Osterrieth Complex Figure Test, 30-minute delay copy condition
76. Wechsler Adult Intelligence Scale digit span total (scaled)
77. Wechsler Adult Intelligence Scale digit span total
78. Wechsler Adult Intelligence Scale digit span (forwards)
79. Wechsler Adult Intelligence Scale digit span (backwards)
80. Hopkins Verbal Learning Test total recall score
81. Hopkins Verbal Learning Test delayed recall score
82. Hopkins Verbal Learning Test recognition score
83. Children's Sustained Attention to Response Task, percent hits
84. Children's Sustained Attention to Response Task, omissions
85. Children's Sustained Attention to Response Task, reaction time
86. Children's Sustained Attention to Response Task, errors
87. Delis-Kaplan Executive Function System test, scanning task
88. Delis-Kaplan Executive Function System test, numbers task
89. Delis-Kaplan Executive Function System test, letters task
90. Delis-Kaplan Executive Function System test, switching task
91. Delis-Kaplan Executive Function System test, correct sorts
92. Delis-Kaplan Executive Function System test, free description
93. Delis-Kaplan Executive Function System test, recognition and description
94. Delis-Kaplan Executive Function System test, combined description
95. Delis-Kaplan Executive Function System test, verbal score
96. Delis-Kaplan Executive Function System test, perceptual score

B. Non-lesion weights in the best model for each dependent variable

The best model for reaction times in naming employed only lesion data (see Table 2). In what follows, we report weights assigned via data perturbation for the non-lesion variables in the best models for the other three dependent variables that we considered. The first table concerns weights on clinical variables (i.e. demographic variables and total lesion volume). The next table concerns behavioural variables, and is presented in the same order as the list of variable descriptions in the last section.

Table S1: Weights on clinical variables where they appear in the best model for a given stimulation response variable.

VARIABLE	NAME %	JUDGE RT
Sex	-0.025	-0.006
Age	-0.048	-0.017
Schooling years	-0.007	0.017
Handedness before stroke	-0.026	-0.011
Handedness after stroke	0.014	-0.095
Time post stroke	-0.064	0.043
Lesion volume	-0.018	0.141

Table S2: Weights on behavioural variables where they appear in the best model for a given stimulation response variable.

TASK	JUDGE %	NAME %	JUDGE RT
CAT_AUDCOMP_TOTAL_of66	-0.025	-0.092	-0.022
CAT_VISCOMP_TOTAL_of62	-0.048	0.005	-0.150
CAT_REPETITION_TOTAL_of74	-0.007	-0.028	0.008
CAT_NAMING_TOTAL_of94	-0.026	-0.029	-0.051
CAT_semanticmemory	0.014	-0.053	-0.134
CAT_verbalfluency_total	-0.064	-0.073	-0.138
CAT_verbalfluency_animals	-0.018	0.006	-0.048
CAT_verbalfluency_S	-0.092	-0.021	-0.173
CAT_recognitionmemory	0.005	0.012	0.020
CAT_gestureobjectuse	-0.028	0.004	-0.162
CAT_arithmatic	-0.029	-0.010	0.011
CAT_comp_spokenwords	-0.053	0.017	-0.133
CAT_comp_writenwords	-0.073	-0.008	-0.204

CAT_comp_spokensentences	0.006	0.016	0.051
CAT_comp_writtensentences	-0.021	0.015	-0.066
CAT_comp_spokenparagraphs	0.012	0.014	-0.003
CAT_wordrepetition	0.004	0.023	0.030
CAT_wordrepetition_1syl	-0.010	-0.017	-0.026
CAT_complexwordrepetition	0.017	0.013	0.070
CAT_nonwordrepetition	-0.008	-0.002	-0.015
CAT_digitstringrepetition	0.016	0.002	-0.004
CAT_sentencerepetition	0.015	0.013	0.020
CAT_namingobjects	0.014	-0.017	0.105
CAT_namingactions	0.023	-0.025	-0.023
CAT_spokenpicturedescription_total	-0.017	-0.011	-0.003
CAT_spokenpicturedescription_appropriateICWS	0.013	-0.030	0.017
CAT_spokenpicturedescription_inappropriateICWS	-0.002	0.020	-0.127
CAT_reading_words	0.002	-0.007	0.009
CAT_reading_complexwords	0.013	-0.005	0.051
CAT_reading_functionwords	-0.017	-0.038	-0.014
CAT_reading_nonwords	-0.025	-0.005	-0.101
CAT_writing_picturenames	-0.011	0.023	-0.124
CAT_writing_todictation	-0.030	0.013	-0.127
PALPA8_nonword_repetition	0.020	-0.015	0.057
PALPA8_nonword_reading	-0.007	-0.003	-0.043
PALPA9_wordrepetition_HIHF	-0.005	0.006	0.042
PALPA9_wordrepetition_HILF	-0.038	-0.016	-0.027
PALPA9_wordrepetition_LIHF	-0.005	0.024	0.108
PALPA9_wordrepetition_LILF	0.023	-0.047	0.071
PALPA9_nonwordrepetition	0.013	0.020	0.102
BNT_spontaneousandstim	-0.015	-0.009	-0.031
BNT_allcues	-0.003	0.007	0.001

BNT_nocues	0.006	0.006	-0.038
CN_correct_CR	-0.016	-0.001	-0.024
CN_correct_SY	0.024	0.017	0.106
CN_correct_SC	-0.047	0.053	-0.170
CN_correct_MR	0.020	0.020	0.080
CN_correct_TOTAL	-0.009	0.014	-0.047
CN_semantic_CE	0.007	-0.005	0.149
CN_semantic_SUP	0.006	-0.003	0.078
CN_semantic_SUB	-0.001	-0.035	0.079
CN_semantic_AE	0.017	-0.020	0.113
CN_semantic_PW	0.053	-0.020	0.149
CN_semantic_CL	0.020	0.016	0.104
CN_semantic_TOTAL	0.014	-0.025	0.164
CN_phonemic_FP	-0.005	0.026	-0.080
CN_phonemic_PE	-0.003	0.004	-0.027
CN_phonemic_PH	-0.035	-0.014	-0.141
CN_phonemic_NE	-0.020	-0.008	-0.122
CN_phonemic_TOTAL	-0.020	0.013	-0.134
CN_omission_IR	0.016	0.012	0.027
CN_omission_NR	-0.025	-0.006	-0.008
CN_omission_TOTAL	0.026	-0.010	0.017
CN_misc_PS	0.004	-0.045	0.028
CN_misc_MX	-0.014	-0.028	0.047
CN_misc_UR	-0.008	0.042	-0.022
CN_misc_SW	0.013	-0.029	-0.052
CN_misc_VE	0.012	-0.020	0.113
CN_misc_TOTAL	-0.006	0.023	-0.006
PPT	-0.010	0.032	-0.134
CATTELS	-0.045	0.013	-0.078

RPM	-0.028	0.000	-0.047
REYS_immediatecopy	0.042	-0.019	0.030
REYS_3mindelaycopy	-0.029	-0.030	-0.157
REYS_30mindelaycopy	-0.020	-0.043	-0.161
WAIS_digitspan_scaledscore	0.023	0.026	-0.015
WAIS_digitspan_total	0.032	-0.038	0.016
WAIS_digitspan_forward	0.013	0.027	0.028
WAIS_digitspan_backwards	0.000	-0.044	-0.016
HVLT_totalrecall	-0.019	-0.049	-0.074
HVLT_delayedrecall	-0.030	-0.015	-0.064
HVLT_recognition	-0.043	0.013	-0.239
CSART_percenthits	0.026	-0.040	0.047
CSART_omissions	-0.038	-0.025	-0.011
CSART_RT	0.027	-0.043	0.050
CSART_errors	-0.044	-0.042	-0.005
DKEFS_TM_scanning	-0.049	-0.048	-0.172
DKEFS_TM_numbers	-0.015	-0.006	-0.119
DKEFS_TM_letters	0.013	-0.035	-0.068
DKEFS_TM_switching	-0.040	0.013	-0.098
DKEFS_CS_correctsorts	-0.025	-0.010	-0.172
DKEFS_CS_freedescription	-0.043	0.002	-0.189
DKEFS_CS_reocgnitionddescription	-0.042	0.006	-0.213
DKEFS_CS_combineddescription	-0.048	0.027	-0.223
DKEFS_CS_verbal	-0.006	-0.008	-0.135
DKEFS_CS_perceptual	-0.035	0.010	-0.216
